# Synthetic Tet-inducible artificial microRNAs targeting β-catenin or HIF-1α inhibit malignant phenotypes of bladder cancer cells T24 and 5637

**DOI:** 10.1038/srep16177

**Published:** 2015-11-06

**Authors:** Yonghao Zhan, Yuchen Liu, Junhao Lin, Xing Fu, Chengle Zhuang, Li Liu, Wen Xu, Jianfa Li, Mingwei Chen, Guoping Zhao, Weiren Huang, Zhiming Cai

**Affiliations:** 1 Key Laboratory of Medical Reprogramming Technology, Shenzhen Second People’s Hospital, The First Affiliated Hospital of Shenzhen University Shenzhen, China; 2 Shantou University Medical College, Shantou 515041, China; 3 School of Life Sciences, Sun Yat-Sen University, Guangzhou 510275, China; 4 Shanghai-MOST Key Laboratory of Health and Disease Genomics, Chinese National Human Genome Centerat Shanghai, Shanghai 200000, Shanghai, China

## Abstract

Ribonucleic acid interference (RNAi) based on microRNA (miRNA) may provide efficient and safe therapeutic opportunities. However, natural microRNAs can not easily be regulated and usually cause few phenotypic changes. Using the engineering principles of synthetic biology, we provided a novel and standard platform for the generation of tetracycline (Tet)-inducible vectors that express artificial microRNAs in a dosage-dependent manner. The vector generates a Pol II promoter-mediated artificial microRNA which was flanked by ribozyme sequences. In order to prove the utility of this platform, we chose β-catenin and HIF-1α as the functional targets and used the bladder cancer cell lines 5637 and T24 as the test models. We found that the Tet-inducible artificial microRNAs can effectively silence the target genes and their downstream genes, and induce anti-cancer effects in the two bladder cancer cell lines. These devices can inhibit proliferation, induce apoptosis, and suppress migration of the bladder cancer cell lines 5637 and T24. The Tet-inducible synthetic artificial microRNAs may represent a kind of novel therapeutic strategies for treating human bladder cancer.

Urothelial bladder cancer is difficult to treat with current therapies such as surgery, radiation therapy, and chemotherapy^1^,^2^. Therefore, it is necessary to develop a more efficient and safer therapeutic method for treating urothelial bladder cancer.

RNA interference (ribonucleic acid interference, RNAi) is a powerful tool that blocks gene expression in mammalian cells by triggering sequence-specific gene degradation during posttranscriptional gene silencing^3^,^4^. It has been reported that natural microRNAs and designed shRNA are useful to treat bladder cancer^5^,^6^. However, microRNAs usually cause few phenotypic changes due to the divergent functions of their target genes^7^. Although shRNA transcribed by RNA polymerase III (Pol III) promoter (e.g., U6 or H1 promoter) can be used to effectively silence gene expression, long-term suppression could be problematic and natural Pol III could not easily be used to directly response to external ligands^8^.

Medical synthetic biology is an emerging field that combines engineering principles with biology to achieve the design and production of new artificial devices for controlling complex biological phenotypes of living organisms^9^,^10^,^11^. Using the engineering principles of medical synthetic biology, we provided a novel and standard platform for the generation of tetracycline (Tet)-inducible vectors that express artificial microRNAs in a dosage-dependent manner^12^. Furthermore, we attached an artificial gene named RGR to the artificial microRNA that, once transcribed, generates an RNA molecule with ribozyme sequences at both ends of the designed artificial microRNA. The primary transcripts of RGR undergo self‐catalyzedcleavage to precisely release the designed artificial microRNA mediated by Pol II promoters^13^.

Our results demonstrated that the Tet-inducible artificial microRNAs targeting β-catenin and HIF-1α can effectively inhibit malignant phenotypes of bladder cancer cells in a dosage-dependent manner.

## Results

### Design and construct the optimized Tet-inducible artificial microRNAs

We set out to construct the Tet-inducible artificial microRNAs that target β-catenin or HIF-1α mRNAs. To silence β-catenin or HIF-1α mRNAs, the RNAi sequence of β-catenin or HIF-1α (shRNA-β-catenin or shRNA-HIF-1α) was used to replace the mature-miR-30 encoding region of pre-miR-30 scaffold. Then we got the “amiRNA β-catenin” and “amiRNA HIF-1α”. Similarly, the negative control sequence was also constructed by using the sequence of shRNA-NC to replace the corresponding region of pre-miR-30 scaffold. Then we linked the artificial microRNA with an artificial gene named RGR and generated an RNA molecule with ribozyme sequences at both ends of the designed artificial microRNA. The primary transcripts of RGR undergo self-catalyzed cleavage to precisely release the designed artificial microRNA. All the related sequences are shown in Table 1. Then we inserted the related sequences into the linearized Tet-on vector (PEV-Lv208) containing RNA Pol II promoter for expression of protein-coding genes and got the Tet-inducible amiRNA systems.

### The effects of Tet-inducible artificial microRNAs on the expression levels of the target genes and their downstream genes in bladder cancer cells

**Figure 1:**
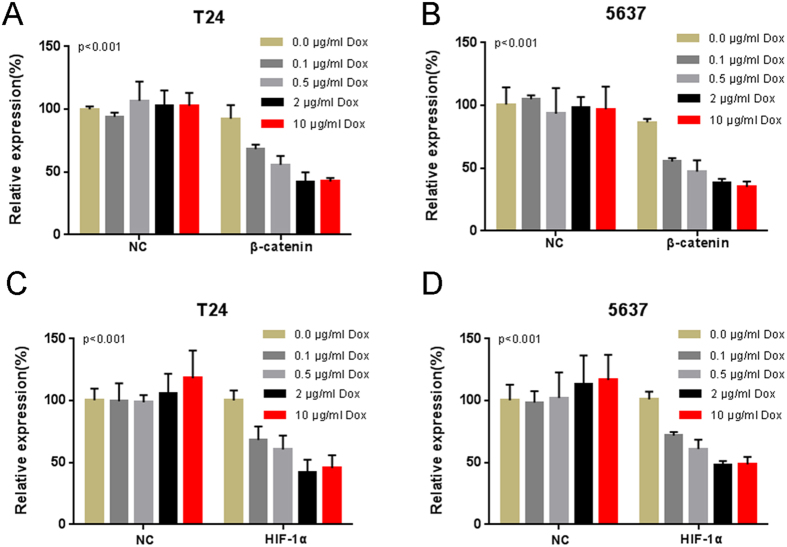
The effects of Tet-inducible artificial microRNAs on the expression levels of their target genes in bladder cancer cells. The relative expression levels of β-catenin (**A**,**B**) and HIF-1α (**C**,**D**) were evaluated using qRT-PCR. Data are shown as mean ± SD.

**Figure 2:**
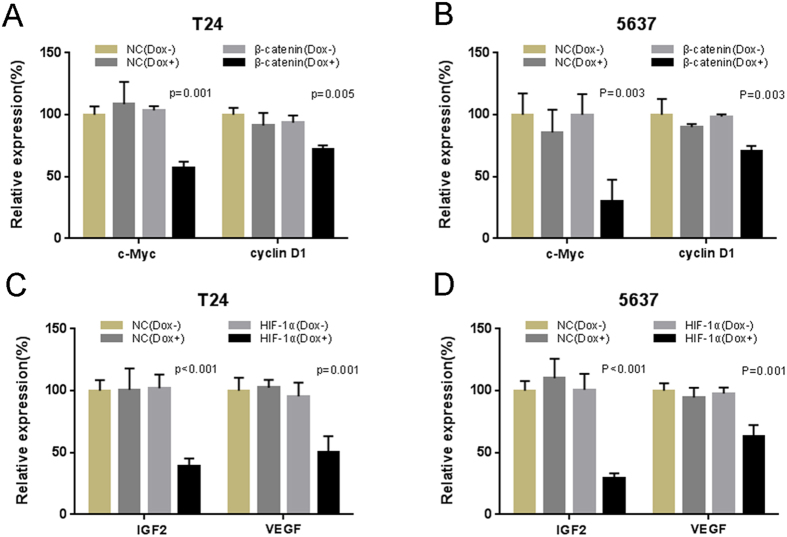
The effects of Tet-inducible artificial microRNAs on the expression levels of the downstream genes in bladder cancer cells. The relative expression levels of c-Myc (**A**,**B**), cyclin D1 (**A**,**B**), IGF2 (**C**,**D**) and VEGF (**C**,**D**) were evaluated using qRT-PCR. Data are shown as mean ± SD.

We transfected the plasmids expressing either the corresponding Tet-inducible artificial microRNA or the negative control into bladder cancer cells, and examined the expression levels of the targeted genes and their downstream genes in these cells. We measured the relative expression level of β-catenin mRNA/HIF-1α mRNA by using qRT-PCR at 48 h post-transfection. The “amiRNA β-catenin” induced by doxycycline could inhibit the expression level of β-catenin mRNA in T24 (Fig. 1A) and 5637 (Fig. 1B). Similarly, the “amiRNA HIF-1α” induced by doxycycline could reduce the expression level of HIF-1α mRNA in T24 (Fig. 1C) and 5637 (Fig. 1D). These results showed substantial dose-dependent repression upon induction with doxycycline. Because both β-catenin and HIF-1α function as transcriptional activators, we wanted to know whether the constructed amiRNAs could inhibit mRNA expression of their downstream genes. We examined the relative gene expression levels in the amiRNAs-transfected bladder cancer cell lines and the mRNA levels of c-Myc (Fig. 2A,B), cyclin D1 (Fig. 2A,B), IGF2 (Fig. 2C,D), and VEGF (Fig. 2C,D) were down-regulated by the corresponding amiRNA in the bladder cell lines.

### The effects of Tet-inducible artificial microRNAs on cell proliferation in bladder cancer cells

**Figure 3:**
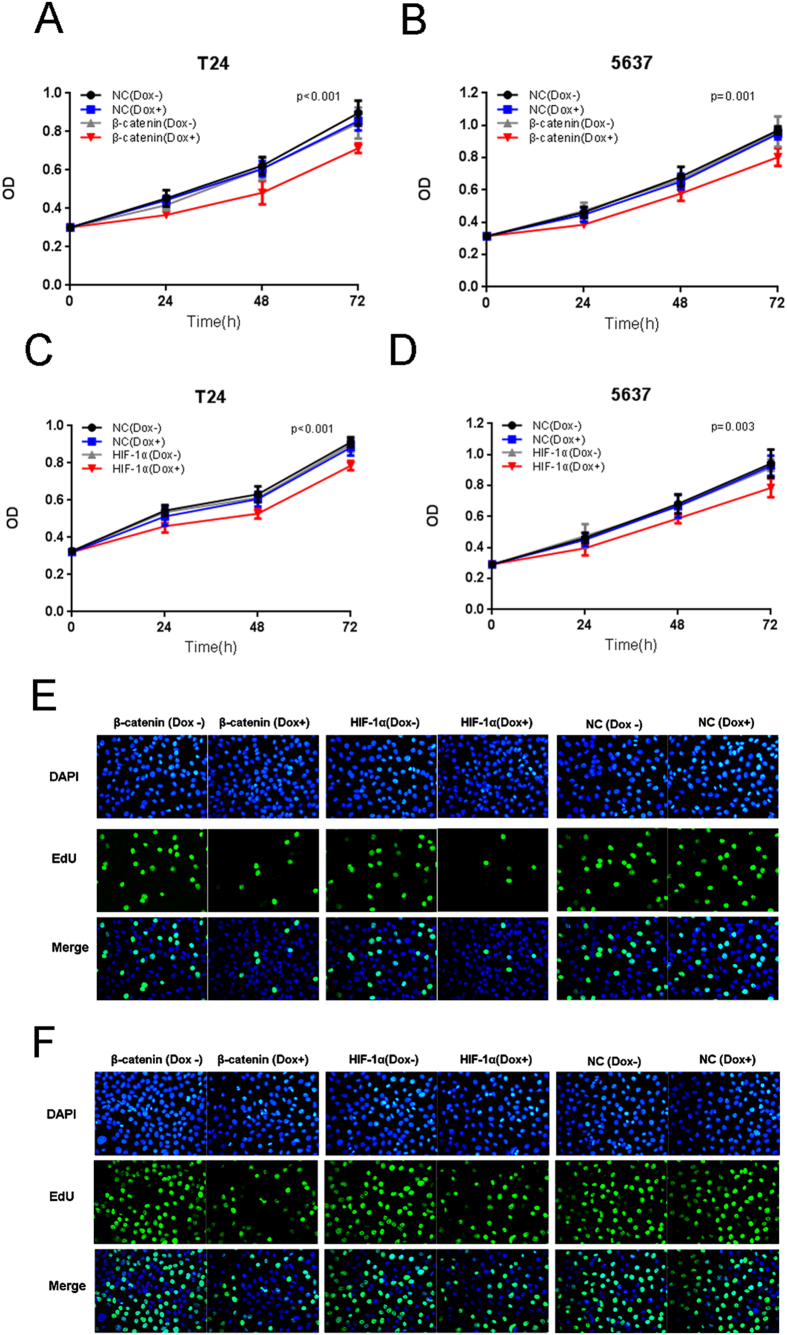
The effects of Tet-inducible artificial microRNAs on cell proliferation in bladder cancer cells. The growth curves of T24/5637 cells treated with “amiRNA β-catenin” (**A**,**B**) or “amiRNA HIF-1α” (**C**,**D**) induced by doxycycline were determined using CCK-8 assay. Data are shown as mean ± SD. The proliferation of T24 (**E**) and 5637 (**F**) treated with “amiRNA β-catenin” or “amiRNA HIF-1α” induced by doxycycline were also determined using EdU incorporation assay.

We transfected the plasmids expressing either the corresponding Tet-inducible artificial microRNA or the negative control into bladder cancer cells, and examined cell wproliferation. In CCK-8 assays, we demonstrated that the “amiRNA β-catenin” induced by doxycycline (Fig. 3A,B) or the “amiRNA HIF-1α” induced by doxycycline (Fig. 3C,D) effectively reduced cell proliferation when compared with the negative control group. EdU incorporation assays were then used as a further study to determine the effects of amiRNAs on proliferation of bladder cancer cell lines. The results showed that amiRNAs induced by doxycycline could inhibit the cancer cell growth remarkably (Fig. 3E,F).

### The effects of Tet-inducible artificial microRNAs on cell apoptosis in bladder cancer cells

**Figure 4:**
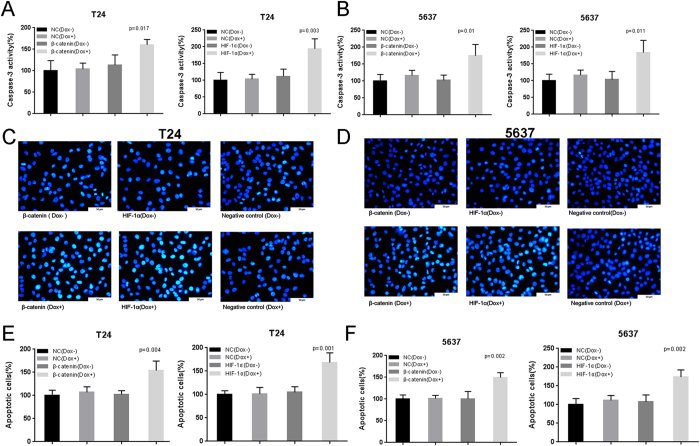
The effects of Tet-inducible artificial microRNAs on cell apoptosis in bladder cancer cells. The relative activity of caspase-3 was calculated in T24/5637 cells treated with “amiRNA β-catenin” (**A**,**B**) or “amiRNA HIF-1α” (**A**,**B**) induced by doxycycline using ELISA assay. The apoptotic cells were observed and calculated in T24/5637 cells treated with “amiRNA β-catenin” (**C**–**F**) or “amiRNA HIF-1α” (**C**–**F**) induced by doxycycline using Hoechst 33258 staining assay. Data are shown as mean ± SD.

We transfected the plasmids expressing the corresponding Tet-inducible artificial microRNA or the negative control into bladder cancer cells, and examined cell apoptosis. The relative activity of caspase-3 was determined using ELISA assay and the apoptosis ratio in bladder cancer cells was measured using Hoechst 33258 staining. The relative activity of caspase-3 were dramatically elevated when cells were treated with the “amiRNA β-catenin” induced by doxycycline (Fig. 4A,B) or the “amiRNA HIF-1α” induced by doxycycline (Fig. 4A,B). These findings were also confirmed by analyzing the apoptosis ratio (Fig. 4C–F).

### The effects of Tet-inducible artificial microRNAs on cell migration in bladder cancer cells

**Figure 5:**
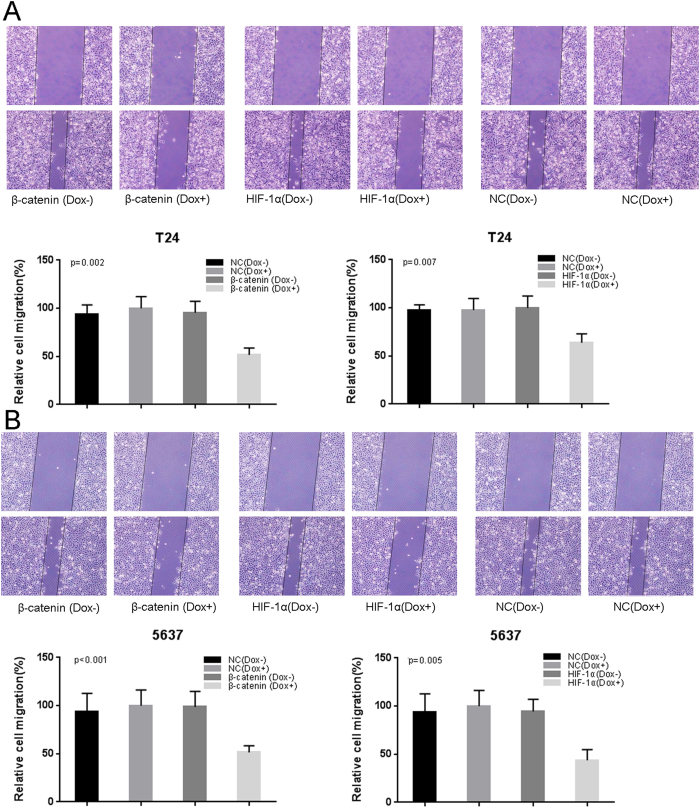
The effects of Tet-inducible artificial microRNAs on cell migration in bladder cancer cells. The relative rate of cell migration was calculated in T24 (**A**) and 5637 (**B**) cells treated with “amiRNA β-catenin” or “amiRNA HIF-1α” induced by doxycycline using wound-healing assay. Data are shown as mean ± SD.

Finally, we treated bladder cancer cells with the Tet-inducible artificial microRNAs and measured cell migration with wound healing assay. Decreased cell motility was observed after transfection of the “amiRNA β-catenin” induced by doxycycline (Fig. 5A,B) or the“amiRNA HIF-1α” induced by doxycycline (Fig. 5A,B).

## Discussion

In the past ten years, the community of medical synthetic biology has begun to make great strides in using synthetic biology principles and methodologies to treat human diseases^14^,^15^,^16^. Furthermore, the basic cancer genetic research has made many remarkable achievements^17^,^18^. Inspired by these exciting works, we proposed that medical synthetic biology can also offer novel solutions to cancer treatment.

Based on the engineering principles and methodologies of synthetic biology, we set out to translate the results of basic cancer research and to build complex devices for treating cancers by using existing genetic parts(such as the promoters, terminators, and shRNA/miRNA expression scaffolds)^19^. The targets of these devices are the cancer-related genes highlighted by the previous works. For example, the gene β-catenin and HIF-1α can be considered to be cancer-related genes and play important roles in the proliferation, apoptosis, and migration of bladder cancer cells by regulating Wnt/β-catenin and HIF-1α pathway, respectively^20^,^21^.

In this study, we constructed the devices (the Tet-inducible artificial microRNAs) and tested their anti-cancer effects. Our results demonstrated that the Tet-inducible artificial microRNAs targeting β-catenin and HIF-1α can be used to effectively silence the cancer-related genes and inhibit malignant phenotypes of bladder cancer cells in a dosage-dependent manner.

In conclusion, we have provided a standard synthetic biology platform for constructing Tet-inducible artificial microRNAs that can silence cancer-related genes in a dosage-dependent manner. This work provides a new approach for quantitatively controlling specific targets in human cancer.

## Materials and Methods

### Cell lines and cell culture

Bladder cancer T24 and 5637 cells used in this study were purchased from the Institute of Cell Research, Chinese Academy of Sciences, Shanghai, China. The 5637 cells were cultured in RPMI-1640 ((Invitrogen, Carlsbad, CA, USA) plus 10% fetal bovine serum. The T24 cells were cultured in DMEM (Invitrogen, Carlsbad, CA, USA) plus 10% fetal bovine serum. Plates were then placed at 37 °C with a humidified atmosphere of 5% CO_2_ in incubator.

### Construction of the optimized Tet-inducible artificial microRNAs

Plasmid vector PEV-Lv208 was purchased from FulenGen, Guangzhou, China. The sequences of the related Tet-inducible artificial microRNAs and the negative control were designed and chemically synthesized. These synthetic related sequences were inserted into PEV-Lv208 vector at the restriction sites of BamHI/XhoI. All these vectors were then transformed into *E. coli*, and the positive clones were identified by electrophoresis and confirmed by Sanger sequencing. Thorough descriptions of the related Tet-inducible artificial microRNA sequences are presented in Table 1.

### Cell transfection

The cells were cultured 24 h prior to transfection. Then, the cells were transiently transfected with the synthetic devices using Lipofectamine 2000 Transfection Reagent (Invitrogen, Carlsbad, CA, USA) according to the manufacturer’s instructions.

### RNA extraction and qRT-PCR

At forty-eight hours post-transfection, 1 × 10^6^ cells were collected and the total RNA of different groups were extracted using the Trizol reagent (Invitrogen, USA) according to the manufacturer’s protocol. The concentration and purity of the total RNA were detected with UV spectrophotometer analysis at 260 nm. Synthesis of cDNA was performed by using SuperScript III^®^ (Invitrogen) according to the manufacturer’s instructions. Quantitative real-time PCR was performed using the ABI PRISM 7000 Fluorescent Quantitative PCR System (Applied Biosystems, Foster City, CA, USA) according to the manufacturer’s instructions. Expression fold changes were calculated using 2^−ΔΔCt^ methods.

### Cell proliferation assay

Cell proliferation was determined using Cell Counting Kit-8 (Beyotime Inst Biotech, China) according to manufacture’s instructions. Briefly, 5 × 10^3^ cells/well were seeded in a 96-well flat-bottomed plate, and grown at 37 °C for 24 h. At 24, 48, 72 hours after transfection, 10 μl of CCK-8 (5 mg/ml) was added to each well of a 96-well plate and the cells were cultured for 1 hour. The absorbance was finally determined at a wavelength of 450 nm using an ELISA microplate reader (Bio-Rad, Hercules, CA, USA). Experiments were repeated at least three times.

Cell proliferation was also determined by Ethynyl-2-deoxyuridine incorporation assay using the corresponding kit (Beyotime Inst Biotech, China). Briefly, 1 × 10^5^ cells/well were seeded in a 96-well flat-bottomed plate, and incubated with 100 μl of 50 μM EdU per well for 2 h. Then, the cells were fixed for 30 min at room temperature by using 50 μl of fixing buffer. After removing the buffer, the cells were incubated with 50 μl of 2 mg/ml glycine for 5 min followed by washing with 100 μl of PBS. The cells in each well were then added with 100 μl of permeabilization buffer followed by washing with 100 μl of PBS. Subsequently, cells were added with 100 μl of 1X Apollo solution for 30 min at room temperature in the dark. After that, cells were incubated with 100 μl of 1X DAPI solution for 30 min at room temperature in the dark followed by washing with 100 μl of PBS. The cells were then observed using fluorescence microscopy.

### Cell apoptosis assay

Cell apoptosis was revealed by both Hoechst 33258 staining assay and ELISA assay. Apoptosis ratio in bladder cancer cells was measured using Hoechst 33258 staining kit (Beyotime, Shanghai, China), and Caspase-3 activity was measured using a Caspase-3 Colorimetric Assay kit (Abcam, Cambridge, UK) according to the manufacturer’s instructions at 48 hours after transfection. Experiments were repeated at least three times in duplicates.

### Cell motility assay

Cell motility was determined by wound-healing assay. A wound field was created using a sterile 200 μl pipette tip in about 90% confluent cells. The migration of cells was monitored with a digital camera system. The cell migration distance (μm) was calculated by the software program HMIAS-2000, at 24 hours after transfection. These experiments were repeated at least three times.

### Statistical analyses

All experimental data from three independent experiments were analyzed by Student’s t-test or ANOVA and P < 0.05 was considered statistically significant. All statistical tests were conducted by SPSS version 19.0 software (SPSS Inc. Chicago, IL, USA).

## Additional Information

**How to cite this article**: Zhan, Y. *et al.* Synthetic Tet-inducible artificial microRNAs targeting β-catenin or HIF-1α inhibit malignant phenotypes of bladder cancer cells T24 and 5637. *Sci. Rep.* **5**, 16177; doi: 10.1038/srep16177 (2015).

## Figures and Tables

**Table 1:** Artificial MicroRNA Sequences in the Vector.

Name	Artificial MicroRNA Sequence
amiRNA β-catenin	TACCTTCTGATGAGTCCGTGAGGACGAAACGAGTAAGCTCGTCAAGGTATATTGCTGTTGACAGTGAGCGCAAACAGTCTTACCTGGACTCTGATAGTGAAGCCACAGATGTATCAGAGTCCAGGTAAGACTGTTTTTGCCTACTGCCTCGGGCCGGCATGGTCCCAGCCTCCTCGCTGGCGCCGGCTGGGCAACATGCTTCGGCATGGCGAATGGGAC
amiRNA HIF-1α	TACCTTCTGATGAGTCCGTGAGGACGAAACGAGTAAGCTCGTCAAGGTATATTGCTGTTGACAGTGAGCGCAGATGCTTACACACAGAAATGATAGTGAAGCCACAGATGTATCATTTCTGTGTGTAAGCATCTTTGCCTACTGCCTCGGGCCGGCATGGTCCCAGCCTCCTCGCTGGCGCCGGCTGGGCAACATGCTTCGGCATGGCGAATGGGAC
amiRNA-NC	TACCTTCTGATGAGTCCGTGAGGACGAAACGAGTAAGCTCGTCAAGGTATATTGCTGTTGACAGTGAGCGCATTCTCCGAACGTGTCACGTATAGTGAAGCCACAGATGTATACGTGACACGTTCGGAGAATTTGCCTACTGCCTCGGGCCGGCATGGTCCCAGCCTCCTCGCTGGCGCCGGCTGGGCAACATGCTTCGGCATGGCGAATGGGAC
