# Author Correction: Synthetic Tet-inducible artificial microRNAs targeting β-catenin or HIF-1α inhibit malignant phenotypes of bladder cancer cells T24 and 5637

**DOI:** 10.1038/s41598-023-42906-4

**Published:** 2023-09-28

**Authors:** Yonghao Zhan, Yuchen Liu, Junhao Lin, Xing Fu, Chengle Zhuang, Li Liu, Wen Xu, Jianfa Li, Mingwei Chen, Guoping Zhao, Weiren Huang, Zhiming Cai

**Affiliations:** 1 Key Laboratory of Medical Reprogramming Technology, Shenzhen Second People’s Hospital, The First Affiliated Hospital of Shenzhen University, Shenzhen, China; 2 Shantou University Medical College, Shantou, 515041 China; 3 School of Life Sciences, Sun Yat-Sen University, Guangzhou, 510275 China; 4 Shanghai-MOST Key Laboratory of Health and Disease Genomics, Chinese National Human Genome Centerat Shanghai, Shanghai, 200000 Shanghai China

Correction to: *Scientific Reports* 10.1038/srep16177, published online 6 November 2015

The original Article contains errors. In Figure 3E the EDU and DAPI images of HIF-1a (Dox+) group are incorrect. The Authors discovered that incorrect images were used for this condition. The corrected Figure 3 and accompanying legend appear below as Figure 1:

**Figure 1:**
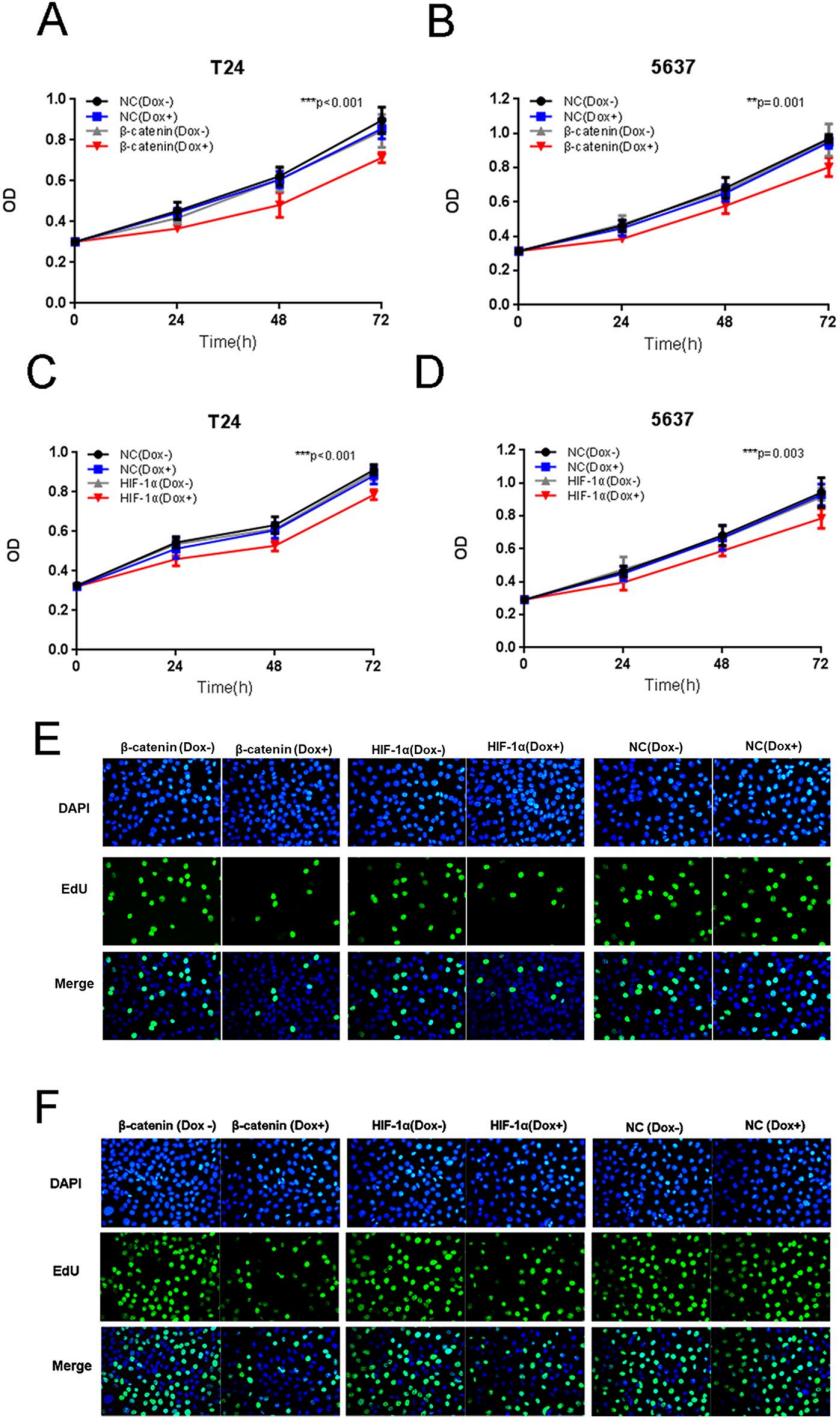
The effects of Tet-inducible artificial microRNAs on cell proliferation in bladder cancer cells. The growth curves of T24/5637 cells treated with “amiRNA β-catenin” (**A**,**B**) or “amiRNA HIF-1α” (**C**,**D**) induced by doxycycline were determined using CCK-8 assay. Data are shown as mean ± SD. The proliferation of T24 (**E**) and 5637 (**F**) treated with “amiRNA β-catenin” or “amiRNA HIF-1α” induced by doxycycline were also determined using EdU incorporation assay.

In Figure 4C the condition beta-catenin (Dox+) is partially overlapping with the condition HF-1alpha(Dox+). The corrected Figure 4 and accompanying legend appear below as Figure 2:

**Figure 2:**
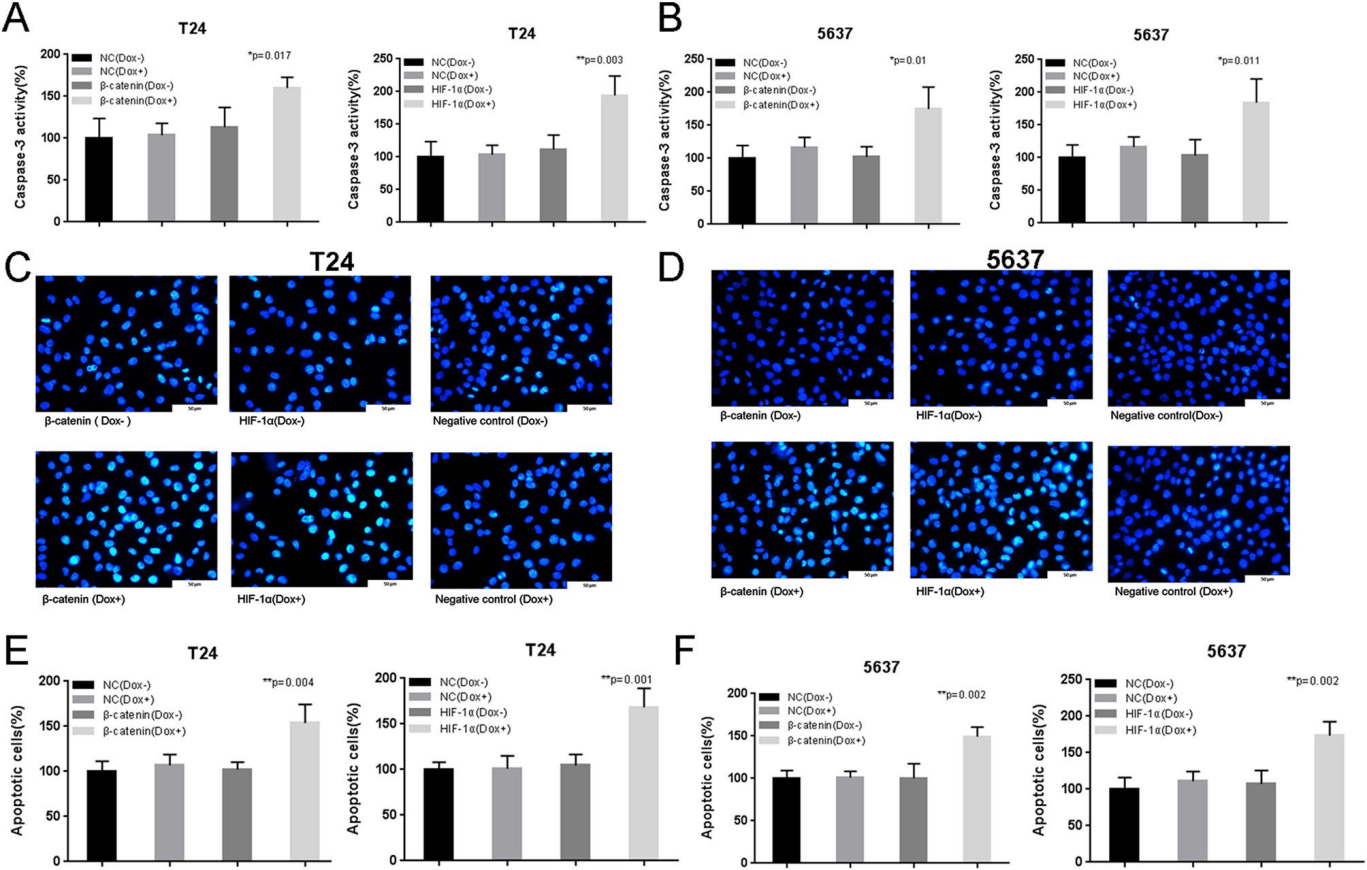
The effects of Tet-inducible artificial microRNAs on cell apoptosis in bladder cancer cells. The relative activity of caspase-3 was calculated in T24/5637 cells treated with “amiRNA β-catenin” (**A**,**B**) or “amiRNA HIF-1α” (**A**,**B**) induced by doxycycline using ELISA assay. The apoptotic cells were observed and calculated in T24/5637 cells treated with “amiRNA β-catenin” (**C**–**F**) or “amiRNA HIF-1α” (**C**–**F**) induced by doxycycline using Hoechst 33,258 staining assay. Data are shown as mean ± SD.

In Figure 5B the conditions beta-catenin (Dox−) (bottom image) and NC (Dox+) (bottom image) seem to be very similar. In Figure 5B the conditions NC (Dox−) (top image) and NC (Dox+) (top image) are partially overlapping. The corrected Figure 5 and accompanying legend appear below as Figure 3:

**Figure 3:**
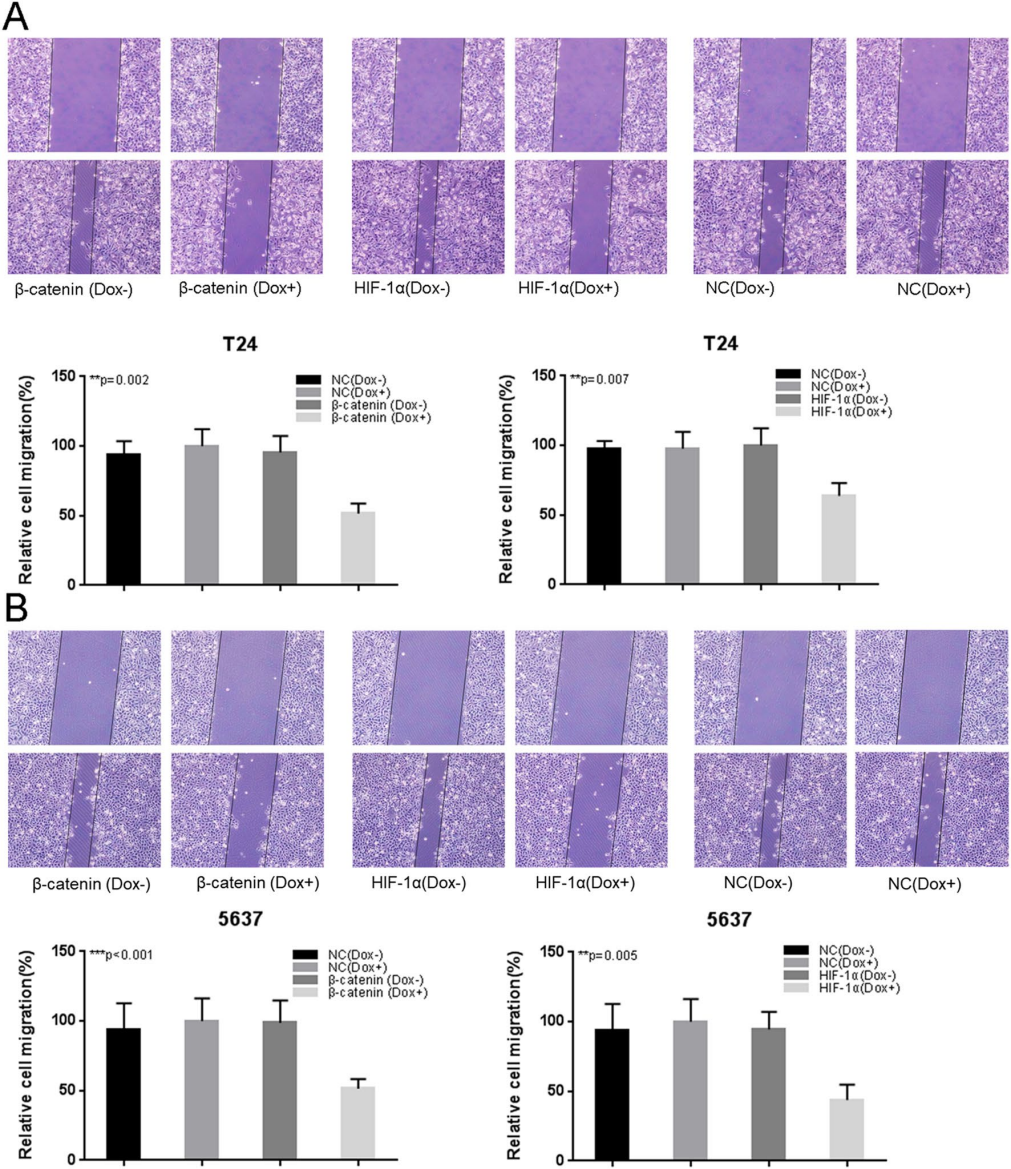
The effects of Tet-inducible artificial microRNAs on cell migration in bladder cancer cells. The relative rate of cell migration was calculated in T24 (**A**) and 5637 (**B**) cells treated with “amiRNA β-catenin” or “amiRNA HIF-1α” induced by doxycycline using wound-healing assay. Data are shown as mean ± SD.

